# Supplementary material for: The ASSIST trial: Acute effects of manipulating strength exercise volume on insulin sensitivity in obese adults: A protocol for a randomized controlled, crossover, clinical trial
Source: PLoS One. 2024 May 28;19(5):e0302480. doi: 10.1371/journal.pone.0302480 (PMC11132464; doi:10.1371/journal.pone.0302480)
Supplement: S2 Table — (DOCX) [file pone.0302480.s002.docx]

**S2 Table**

**WHO Trial Registration Data Set**

| **Data category** | **Information** |
| --- | --- |
| **Primary Registry and Trial Identifying Number** | ReBEC #RBR-3vj5dc5 (<https://ensaiosclinicos.gov.br/rg/RBR-3vj5dc5>) |
| **Date of Registration in Primary Registry** | Feb, 8^th^ 2023 |
| **Secondary Identifying Numbers** | World Health Organization Universal Trial Number (UTN): U1111-1287-9212 |
| **Source(s) of Monetary or Material Support** | Federal University of the Jequitinhonha and Mucuri Valleys |
| **Primary Sponsor** | Federal University of the Jequitinhonha and Mucuri Valleys |
| **Secondary Sponsor(s)** | National Council for Scientific and Technological Development (CNPQ) and Minas Gerais State Agency for Research and Development (FAPEMIG) |
| **Contact for Public Queries** | Dr Magalhães: [fcm@unm.edu](mailto:fcm@unm.edu) |
| **Contact for Scientific Queries** | Dr Magalhães: [fcm@unm.edu](mailto:fcm@unm.edu) |
| **Public Title** | How Much Strength Exercise does it take to Improve Insulin Sensitivity? |
| **Scientific Title** | Acute effects of manipulating strength exercise volume on insulin sensitivity in obese adults: a protocol for a randomized controlled, crossover, clinical trial |
| **Countries of Recruitment** | Brazil |
| **Health Condition(s) or Problem(s) Studied** | Obesity |
| **Intervention(s)** | Active: High- and low-volume strength exercise session |
|  | Comparator: control day without exercise |
| **Key Inclusion and Exclusion Criteria** | Inclusion criteria: Individuals of both sexes with obesity (body mass index – BMI > 30 kg/m²); and with central obesity (waist circumference > 102 cm in men and > 88 cm in women); aged over 40 years; with stable body mass (<3 kg) in the last 3 months; able to perform physical activity  Exclusion criteria: Individuals with signs, symptoms or presence of diabetes or any other metabolic disease, cardiovascular disease, cerebrovascular disease, kidney disease, respiratory disease, and osteoarticular disease; use of medication that affect blood glucose or insulin levels; use of anabolic steroids; use of dietary supplements that enhance physical performance (beta-alanine, sodium bicarbonate, caffeine, creatine); pregnant women |
| **Study Type** | Interventional |
|  | Allocation: randomized controlled, crossover, 3-way, statistician-blind |
|  | Primary purpose: prevention |
| **Date of First Enrollment** | March 9th 2023 |
| **Sample Size** | 14 (fourteen) |
| **Recruitment Status** | Not recruiting |
| **Primary Outcome(s)** | Statistically significant change in the mean difference among groups after treatments in the result of indexes of insulin sensitivity calculated from the glucose and insulin results during the glucose tolerance test. |
| **Key Secondary Outcomes** | Secondary outcomes are not expected. |
| **Ethics Review** | Approved |
| **Completion date** | NA |
| **Summary Results** | NA |
| **IPD sharing statement** | Deidentified research data will be made publicly available when the study is completed and published. |
