# Supplementary material for: The ASSIST trial: Acute effects of manipulating strength exercise volume on insulin sensitivity in obese adults: A protocol for a randomized controlled, crossover, clinical trial
Source: PLoS One. 2024 May 28;19(5):e0302480. doi: 10.1371/journal.pone.0302480 (PMC11132464; doi:10.1371/journal.pone.0302480)
Supplement: S1 File — (DOCX) [file pone.0302480.s004.docx]

**ENCLOSED OPINION OF CEP**

**RESEARCH PROJECT DATA**

**Research Title:** Acute effects of manipulating the volume of strength exercises on sensitivity

to insulin in obese adults

**Researcher:** Flávio de Castro Magalhães

**Thematic Area:**

**Version:** 4

**CAAE:** 63190422.0.0000.5108

**Proponent Institution**: Federal University of Jequitinhonha and Mucuri Valleys

**Main Sponsor**: Own Financing

**OPINION DATA**

**Opinion number: 5,873,060**

**Project presentation:**

The information listed here was taken from the Basic Research Information file (PB_Informações_Básicas_do_projeto_2014038, dated 01/25/2023):

Summary:

The beneficial effects of strength training on insulin sensitivity have been increasingly recognized. Interestingly, a wealth of evidence points to the beneficial effects of a single acute bout of resistance exercise on improving insulin sensitivity. However, some questions remain: What would be the minimum volume of strength exercise needed to improve insulin sensitivity? International associations recommend one to three sets per strength exercise to improve overall health, with no clarity in the literature whether this variation in training volume affects the improvement in insulin sensitivity. Thus, the aim of the study will be to investigate the variation in the volume of strength exercise on the improvement of insulin sensitivity. To achieve these goals, we will invite men and women aged 40 years or older, with a body mass index >30 kg/m2 and central obesity (waist circumference >88 cm for women and >102 cm for men). These participants' characteristics will ensure low insulin sensitivity and a greater probability of observing positive effects of resistance exercise. They will perform 3 sessions: 1) high volume – 7 exercises, 8RM intensity, high volume (3 sets per exercise); 2) low volume – same as session 1, but with 1 series per exercise; 3) control – same as session 1, but without performing any exercise. The sessions will be held in the late afternoon, between 8:00 pm and 9:00 pm, and the next morning, between 7:00 am and 8:00 am (between 10 and 11 hours after), the oral glucose tolerance test will be performed ( in a local clinical laboratory), during which blood glucose and insulin concentrations will be measured and from these measurements various indices of insulin sensitivity will be derived. Each trial day will be separated by at least 7 days.

Hypothesis:

The hypothesis of the study is that a session of strength exercises performed close to mechanical failure with high volume will improve insulin sensitivity, while a session with the same characteristics, but of low volume, will lead to a smaller improvement, but still significant.

Proposed Methodology:

Recruitment. Promotion will be through social media, posters around the city and word of mouth. Pre-participation assessments. Participants will answer an anamnesis to assess possible risks that prevent the implementation of the protocol (RIEBE et al., 2015) (appendix 1). Familiarization and strength test. THE FAMILIARIZATION WILL BE PERFORMED IN 4 SEPARATE DAYS FOR AT LEAST 48 HOURS, always performing 8 repetitions. On the first day, the practitioner will be instructed to lift a light load, in which performing 8 repetitions is considered “easy” according to the OMNI-RES scale (ROBERTSON et al., 2003). In the second session, a load considered “a little difficult” should be lifted. In the third session, considered “difficult”. In the 4th session, between “difficult” and “extremely difficult”, mimicking the effort expected in the strength test. On another day, participants will perform the 8 RM test (Taylor & Fletcher, 2012). Food control. ON THE DAY PRIOR TO SESSION 1, PARTICIPANTS WILL BE ADVISED TO WRITE THE FOOD INTAKE IN A DIARY SO THAT THEY REPEAT THE SAME INGESTION ON THE DAYS PRIOR TO SESSIONS 2 AND 3. ON THE DAY OF SESSION 1, BEFORE REPORTING TO THE LABORATORY, THEY MUST DO THE SAME: WRITE DOWN WHAT AND AT THE MOMENT THEY DRINK, SO THAT THEY REPEAT IN THE OTHER 2 DAYS (ANNEX 2). ONE HOUR BEFORE THE SESSIONS, THEY SHOULD EAT A STANDARDIZED MEAL (60% CARBOHYDRATE, 25% FAT AND 15% PROTEIN; 200-250 kCAL, DEPENDING ON THE PARTICIPANT'S NEEDS) PRESCRIBED BY THE UFVJM NUTRITION TEAM. IMMEDIATELY AFTER THE EXERCISE SESSIONS, A STANDARD SNACK WILL BE OFFERED (60% CARBOHYDRATE, 25% FAT AND 15% PROTEIN; 200-250 KCAL, DEPENDING ON THE PARTICIPANT'S NEEDS), WHICH MUST BE INTAKED UP TO 15 MIN AFTER THE SESSION, AND WILL BE A STANDARD DINNER IS OFFERED, MUST BE CONSUMED BETWEEN 9:30 PM AND 10:00 PM THE SAME DAY (60% CARBOHYDRATE, 25% FAT AND 15% PROTEIN; 450-600 KCAL, DEPENDING ON THE PARTICIPANT'S NEEDS). Experimental situations. 3 experimental sessions will be held randomly separated by at least 7 days: 1) Session 1 (high volume). Participants will report to the academy at 4:30 pm. From 17:00h the experimental session will be held: 7 strength exercises: squat with hexagonal bar; 2 – Straight barbell bench press; 3 – Horizontal Leg Press; 4- Pulled forward with bar; 5 – Extension chair; 6 – Machine shoulder development; 7 – Flex chair. PARTICIPANTS WILL PERFORM 3 SERIES WITH A LOAD OF 8RM, WITH THE MAXIMUM OF REPETITIONS PER SERIES UNTIL MOMENTARY FATIGUE. Each repetition will consist of 1 sec in the concentric phase and 2 sec in the eccentric phase (controlled with the aid of a metronome). IT WILL BE EXPLAINED IN MINUTES TO THE PARTICIPANTS DURING THE FAMILIARIZATION. THERE WILL BE A REST OF 90 TO 120S BETWEEN THE SERIES AND BETWEEN THE EXERCISES. AT THE END OF EACH SERIES, THEY WILL ANSWER THE OMNI-RES SCALE, IN ORDER TO CONFIRM THAT THE NUMBER OF REPETITIONS IN RESERVE WAS ZERO. 2) Session 2 (low volume) – Procedures will be identical to session 1, with the exception of the number of series performed: 1 series. 3) Session 3 (control) – Procedures will be identical to session 1, with the exception of performing strength exercises. In order to simulate all the other procedures performed on the team, they will just sit on the equipment for the same amount of time as the high volume session. Oral Glucose Tolerance Test. PARTICIPANTS WILL SHOW UP AT A LOCAL LABORATORY (EMÍLIO AVELAR LABORATORY – CLINICA CENTERMED - ADDRESS: RUA DO FOGO, 400) BETWEEN 07:00 AND 08:00 AM FOLLOWING THE SESSIONS. A catheter will be inserted into the antecubital vein, and a blood sample will be collected (min 0). Then they ingest 75 grams of glucose in 300 ml of water and blood samples are taken every 30 minutes up to 120 minutes, totaling 5 withdrawals. Plasma concentrations of glucose and insulin will be analyzed.

Inclusion Criteria:

Insulin resistance has been related to increased lipotoxicity, a consequence of an increase in the amount of body fat (Yazc & Sezer, 2017). Thus, the inclusion criteria are individuals of both sexes with obesity (body mass index - BMI > 30 kg/m2), and with central obesity (waist circumference > 102 cm in men and > 88 cm in women), over 40 years old, WITH STABLE BODY MASS (<3 KG) IN THE LAST 3 MONTHS. PARTICIPANTS MUST ALSO BE ABLE TO PERFORM PHYSICAL ACTIVITY, WHICH WILL BE ASSESSED BY THE ANSWERS GIVEN DURING THE ANAMNESIS (ANNEX 1).

Exclusion Criteria:

EXCLUSION CRITERIA ARE INDIVIDUALS WITH SIGNS, SYMPTOMS OR PRESENCE OF DIABETES OR ANY OTHER METABOLIC DISEASE, CARDIOVASCULAR DISEASES, CEREBROVASCULAR DISEASES, KIDNEY DISEASES, RESPIRATORY DISEASES, AND OSTEOARTICULAR DISEASES (RIEBE ET AL., 2015). IN ADDITION, THOSE WHO REPORT THE USE OF ANY DRUG THAT MAY INFLUENCE THE EXPECTED RESULTS AND THE USE OF ANABOLIC STEROIDS WILL BE EXCLUDED.

**Purpose of Research:**

The information listed here was taken from the Basic Research Information file (PB_Informações_Básicas_do_projeto_2014038, dated 01/25/2023):

Primary Purpose:

Main goal

The overall objective will be to investigate whether the volume of resistance exercise affects insulin sensitivity observed acutely, that is, from a single session, in obese adults.

Secondary Purpose:

Specific objectives

THE SPECIFIC OBJECTIVES WILL BE TO EVALUATE THE ACUTE INFLUENCE OF 7 EXERCISES OF LARGE MUSCLE GROUPS PERFORMED IN 3 OR 1 SETS PER EXERCISE, PERFORMED UNTIL MOMENTARY FATIGUE, IN OBESE ADULTS ON:

1. GLUCOSE AND FASTING INSULIN;

2. INSULIN RESISTANCE BY THE INSULIN RESISTANCE HOMEOSTASIS MODEL (HOMA-IR);

3. INSULIN SENSITIVITY USING THE QUANTITATIVE INSULIN SENSITIVITY SCREENING INDEX;5. THE MATSUDA INSULIN SENSITIVITY INDEX;

6. THE CEDERHOLM ́S INSULIN SENSITIVITY INDEX;

7. THE INSULIN MUSCLE SENSITIVITY INDEX;

8. THE GLUCOSE-STIMULATED INSULIN SENSITIVITY INDEX; 9. THE ORAL DISPOSITION INDEX;

10. THE GUTT INDEX;

11. THE AVIGNON ET AL. TABLE OF CONTENTS;

12. BELFIORE ET AL. INDEX;

13. INDEX BY STUMVOLL ET AL.;

14. MCAULEY ET AL. INDEX;

15. THE AREA UNDER THE GLUCOSE AND INSULIN CURVE.

**Assessment of Risks and Benefits:**

The information listed here was taken from the Basic Research Information file (PB_Informações_Básicas_do_projeto_2014038, dated 01/25/2023):

Risks:

Embarrassment. There are risks of discomfort and embarrassment during the application of the anamnesis and completion of the food record. They will be minimized as the participant may fail to answer any question. In addition, they will be applied in a reserved room, individually, where only one member of the team and the participant will be present. Identification. There is a risk of identifying participants in the anamnesis and food recall. They will be minimized because only the researcher coordinating the project will have access to the answers to the documents that will be kept in his workroom, locked inside a cabinet that only he holds the key, and after the completion of the research they will be destroyed. Oral glucose tolerance test. There are risks related to the oral glucose tolerance test. Due to the insertion of the catheter in the antecubital vein and blood collection, the main risks are weakness, slight malaise, sweating (increased sweat production), dizziness, pressure drop, hematoma (purplish), pain, infection, and possibility of discomfort during blood collection. These risks will be minimized by the following procedures: asepsis before collection, placement of an ice pack, use of disposable materials, performance of the procedure by a trained technician. Ingesting the glucose solution can bring risks related to nausea, dizziness, weakness, diarrhea, tachycardia, tremors and sweating (increased sweat production). These risks will be minimized by carrying out the test in an analysis laboratory specialized in procedures of this nature, where there are adequate conditions to deal with any intercurrence reported above, such as placing the participant on a stretcher and the presence of a nearby toilet. Anthropometric measurements. There are risks in carrying out anthropometric measurements, such as embarrassment during the measurement of body mass, height and waist circumference. To minimize the risk of embarrassment, this assessment will only be carried out in the presence of a team member in a private room. Cardiovascular events. To minimize the risk of cardio or cerebrovascular events, participants will respond to the anamnesis and if signs/symptoms of diseases are detected, whose repercussions may cause risk, they will be excluded from the study. Also, if adverse events occur during the sessions, team members are trained in how to proceed with first aid care. Strength exercise. There are risks when performing strength exercises related to musculoskeletal injuries. In order to prevent injuries, participants will be instructed on the correct way to perform the exercises, will have familiarization sessions and will be accompanied in all sessions by a professional trained in Physical Education.

Benefits:

Direct benefit: The oral glucose tolerance test is an important index for the diagnosis of pre-diabetes and diabetes, so it can help participants to better understand their metabolic control. PARTICIPATING IN THIS SURVEY MAY MOTIVATE PARTICIPANTS TO BECOME PHYSICALLY ACTIVE. Indirect benefit: PUBLISHED RESULTS FROM RESEARCH MAY CHANGE RECOMMENDATIONS OF STRENGTH EXERCISES FOR OBESE INDIVIDUALS LOOKING TO IMPROVE THEIR HEALTH.

**Comments and Considerations about the Research:**

The information listed here was taken from the Basic Research Information file (PB_Informações_Básicas_do_projeto_2014038, dated 01/25/2023):

Data Analysis Methodology:

WITH THE RESULTS OF THE GLUCOSE TOLERANCE TEST, INSULIN AND GLUCOSE AT MINUTE 0 (FASTING VALUES) WILL BE USED TO CALCULATE INSULIN RESISTANCE WILL BE CALCULATED FROM THE INSULIN RESISTANCE HOMEOSTASIS MODEL (HOMA-IR), USING THE FORMULA GLUCOSE (MMOL) X INSULIN ( μU/ML) ÷ 22.5 (MATTHEWS ET AL., 1985), AND INSULIN SENSITIVITY USING THE QUANTITATIVE INSULIN SENSITIVITY SCREENING INDEX (KATZ ET AL., 2000A) . VARIOUS INDEX OF DERIVATIVES OF THIS TEST (PATARRÃO ET AL., 2014A), SUCH AS THE INSULIN SENSITIVITY INDEX TO ORAL GLUCOSE (MARI ET AL., 2001A) INSULIN SENSITIVITY INDEX OF MATSUDA (MATSUDA & DEFRONZO, 1999A) AND CEDERHOLM ́S (CEDERHOLM & WIBELL, 1990A), INSULIN MUSCLE SENSITIVITY INDEX (ABDULGHANI ET AL., 2007A; MATOS ET AL., 2018), GLUCOSE-STIMULATED INSULIN SENSITIVITY INDEX (MALIN ET AL., 2013B), ORAL DISPOSITION (ABDUL-GHANI ET AL., 2006; MIYAZAKI ET AL., 2008), GUTT INDEX (GUTT ET AL., 2000), AVIGNON ET AL. (COBELLI ET AL., 1987), INDEX OF BELFIORE ET AL. (MONZILLO & HAMDY, 2003a), INDEX BY STUMVOLL ET AL. (STUMVOLL ET AL., 2001a), MCAULEY ET AL. (MCAULEY ET AL., 2001a). IN ADDITION, THE AREA UNDER THE GLUCOSE AND INSULIN CURVE WILL BE CALCULATED USING THE TRAPEZOIDAL METHOD (ISMAIL ET AL., 2019). Data will be expressed as mean and standard deviation, with a confidence interval of 95%. For the analysis of data normality, we will perform the Shapiro-Wilk test. And for normally distributed data, analysis of variance will be used with a source of variation (experimental situation). If a significant main effect is observed, post-hoc Tukey will be used. For non-parametric data, the Kruskal-Wallis test will be used, or Friedman's test, when necessary. The effect size will be calculated and interpreted as follows: 0.2 = low effect, 0.5 = medium effect, and greater than 0.8 = high effect (Ferguson, 2009; Sullivan & Feinn, 2012). The significance level will be 5%. The Prisma program (GraphPad Software, San Diego, CA-USA – version 9.3.1) will be used to analyze the results. All data analysis work will remain blind until completion.

**Considerations about the Mandatory Presentation Terms:**

See field: "Conclusions and Pending Issues and List of Inadequacies"

**Recommendations:**

See field: "Conclusions and Pending Issues and List of Inadequacies"

**Conclusions or Pending Issues and List of Inadequacies:**

Letter of consent attached. Project Approved.

**Final Considerations at the CEP's discretion:**

- According to Circular Letter no. 003/2011/CONEP/CNS, of 03/21/11, when obtaining the TCLE, it is mandatory to initial it on all pages of the same, by the research subject or his/her person in charge and by the researcher. The responsible researcher must affix his signature on the last page of the referred term.

- The final report must be presented to the CEP at the end of the study. Research discontinued without justification accepted by the CEP that approved it is considered unethical.

- If there are any intercurrences during the execution of the research project, it is the responsibility of the responsible researcher to communicate it through an amendment to the CEP via Plataforma Brasil. Research with modifications to its previously approved initial protocol without justification accepted by the CEP that approved it is considered unethical.

The project complies with the ethical precepts for research involving human beings set out in Resolution 466/12 CNS.

**OPINION STATUS**: APPROVED

**Requires CONEP Appraisal**: No

DIAMANTINA, February 2, 2023

**Signed by:**

**FABIO LUIZ MENDONÇA MARTINS (Coordinator)**
