# Supplementary material for: The ASSIST trial: Acute effects of manipulating strength exercise volume on insulin sensitivity in obese adults: A protocol for a randomized controlled, crossover, clinical trial
Source: PLoS One. 2024 May 28;19(5):e0302480. doi: 10.1371/journal.pone.0302480 (PMC11132464; doi:10.1371/journal.pone.0302480)
Supplement: S2 File — (DOCX) [file pone.0302480.s005.docx]

**PARECER CONSUBSTANCIADO DO CEP**

**DADOS DO PROJETO DE PESQUISA
Título da Pesquisa:** Efeitos agudos da manipulação do volume de exercícios de força sobre a sensibilidade à insulina em adultos obesos

**Pesquisador:** Flávio de Castro Magalhães

**Área Temática:
Versão:** 4
**CAAE:** 63190422.0.0000.5108
**Instituição Proponente:**Universidade Federal dos Vales do Jequitinhonha e Mucuri

**Patrocinador Principal:** Financiamento Próprio

**DADOS DO PARECER**

**Número do Parecer:** 5.873.060

**Apresentação do Projeto:**

As informações aqui elencadas foram retiradas do arquivo Informações Básicas da Pesquisa (PB_Informações_Básicas_do_projeto_2014038, de 25/01/2023):

Resumo:
Os efeitos benéficos do treinamento de força sobre a sensibilidade à insulina têm sido cada vez mais reconhecidos. Interessantemente, diversas evidências apontam para os efeitos benéficos de uma única sessão aguda de exercício de força sobre a melhora da sensibilidade à insulina. No entanto, algumas dúvidas permanecem: Qual seria o volume mínimo necessário de exercício de força para melhorar a sensibilidade à insulina? Associações internacionais recomendam de uma a três séries por exercício de força para a melhora na saúde em geral, não havendo clareza na literatura se essa variação no volume de treino afeta a melhora na sensibilidade à insulina. Assim, o objetivo do estudo será investigar a variação do volume de exercício de força sobre a melhora da sensibilidade à insulina. Para alcançar esses objetivos, iremos convidar homens e mulheres de 40 anos ou mais, com índice de massa corporal >30 kg/m2 e obesidade central (circunferência da cintura >88 cm para mulheres e >102 cm para homens). Essas caraterísticas dos participantes garantirão baixa sensibilidade à insulina e maior probabilidade de observarmos efeitos positivos do exercício de força. Eles realizarão 3 sessões: 1) alto volume – 7 exercícios, intensidade de 8 RM, alto volume (3 séries por exercício); 2) baixo volume – mesmo que sessão 1, porém com 1 série por exercício; 3) controle – mesmo que sessão 1, porém sem a realização de nenhum exercício. As sessões serão realizadas no final da tarde, entre 20:00 e 21:00, e na manhã seguinte, entre 07:00 e 08:00 (entre 10 a 11 horas após), será realizado o teste de tolerância oral à glicose (em laboratório clinico local), durante o qual serão medidas as concentrações sanguíneas de glicose e insulina e dessas medidas, serão derivados diversos índices de sensibilidade à insulina. Cada dia experimental será separado por pelo menos 7 dias.

Hipótese:
A hipótese do estudo é que uma sessão de exercícios de força realizados até próximos da falha mecânica com alto volume irá melhorar a sensibilidade à insulina, enquanto uma sessão com as mesmas caraterísticas, mas de baixo volume, irá levar a melhora menor, mas ainda assim significativa.

Metodologia Proposta:
Recrutamento. A divulgação será através de mídias sociais, cartazes pela cidade e boca a boca. Avaliações pré-participação. Os participantes responderão uma anamnese pra avaliar possíveis riscos que impeçam a realização do protocolo (RIEBE et al., 2015) (anexo 1). Familiarização e teste de força. SERÁ REALIZADA A FAMILIARIZAÇÃO EM 4 DIAS SEPARADOS POR PELO MENOS 48 HORAS, sempre realizando 8 repetições. No primeiro dia o praticante será orientado a levantar uma carga leve, na qual realizar 8 repetições seja considerado “pouco fácil” de acordo com a escala OMNI-RES (ROBERTSON et al., 2003). Na segunda sessão, deverá ser levantada carga considerada “um pouco difícil”. Na terceira sessão, considerada “difícil”. Já na 4a sessão, entre “difícil” e “extremamente difícil”, mimetizando o esforço esperado no teste de força. Em outro dia os participantes realizarão o teste de 8 RM (Taylor & Fletcher, 2012). Controle alimentar. NO DIA ANTERIOR À SESSÃO 1, OS PARTICIPANTES SERÃO ORIENTADOS A ANOTAR EM UM DIÁRIO A INGESTÃO ALIMENTAR PARA QUE REPITAM A MESMA INGESTÃO NOS DIAS ANTERIORES ÀS SESSÕES 2 E 3. NO DIA DA SESSÃO 1, ANTES DE SE REPORTAREM AO LABORATÓRIO, DEVERÃO FAZER O MESMO: ANOTAR O QUE E EM MOMENTO INGERIRAM, PARA QUE NOS OUTROS 2 DIAS ELES REPITAM (ANEXO 2). UMA HORA ANTES DAS SESSÕES, DEVERÃO INGERIR UMA REFEIÇÃO PADRONIZADA (60% CARBOIDRATO, 25% GORDURA E 15% PROTEÍNA; 200-250 kCAL, A DEPENDER DA NECESSIDADE DE PARTICIPANTE) PRESCRITA PELA EQUIPE DE NUTRIÇÃO DA UFVJM. LOGO APÓS AS SESSÕES DE EXERCÍCIOS SERÁ OFERTADO UM LANCHE PADRÃO (60% CARBOIDRATO, 25% GORDURA E 15% PROTEÍNA; 200-250 KCAL, A DEPENDER DA NECESSIDADE DE PARTICIPANTE), QUE DEVERÁ SER INGERIDO ATÉ 15 MIN APÓS A SESSÃO, E SERÁ OFERECIDA UMA JANTA PADRÃO, DEVENDO SER CONSUMIDA ENTRE 21:30 E 22:00H DAQUELE MESMO DIA (60% CARBOIDRATO, 25% GORDURA E 15% PROTEÍNA; 450-600 KCAL, A DEPENDER DA NECESSIDADE DE PARTICIPANTE). Situações experimentais. Serão realizadas 3 sessões experimentais de forma aleatória separadas por no mínimo 7 dias: 1) Sessão 1 (alto volume). Os participantes se reportarão à academia às 16:30h. A partir das 17:00h a sessão experimental será realizada: 7 exercícios de força: agachamento com barra hexagonal; 2 – Supino reto com barra; 3 – Leg Press horizontal; 4- Puxada a frente com barra; 5 – Cadeira extensora; 6 – Desenvolvimento de ombro na máquina; 7 – Cadeira flexora. OS PARTICIPANTES IRÃO REALIZAR 3 SÉRIES COM A CARGA DE 8RM, COM O MÁXIMO DE REPETIÇÕES POR SÉRIE ATÉ A FADIGA MOMENTÂNEA. CADA REPETIÇÃO CONSISTIRÁ DE 1 SEG NA FASE CONCÊNTRICA E 2 SEG NA FASE EXCÊNTRICA (CONTROLADA COM AUXÍLIO DE UM METRÔNOMO), SENDO A FADIGA MOMENTÂNEA DEFINIDA COMO A INCAPACIDADE DO INDIVÍDUO EM MANTER A DURAÇÃO DA FASE CONCÊNTRICA OU EXCÊNTRICA EM 2 REPETIÇÕES CONSECUTIVAS, O QUE SERÁ MINUCIOSAMENTE EXPLICADO AOS PARTICIPANTES DURANTE A FAMILIARIZAÇÃO. HAVERÁ DESCANSO DE 90 A 120S ENTRE AS SÉRIES E ENTRE OS EXERCÍCIOS. AO FIM DE CADA SÉRIE, RESPONDERÃO A ESCALA OMNI-RES, A FIM DE CONFIRMAR QUE O NÚMERO DE REPETIÇÕES EM RESERVA ERA ZERO. 2) Sessão 2 (baixo volume) – Procedimentos serão idênticos à sessão 1, com exceção do número de séries realizadas: 1 série. 3) Sessão 3 (controle) – Procedimentos serão idênticos à sessão 1, com exceção da execução dos exercícios de força. A fim de simular todos os demais procedimentos realizados na irão apenas sentar-se nos equipamentos durante o mesmo tempo que a sessão de alto volume. Teste de Tolerância Oral à Glicose. OS PARTICIPANTES SE APRESENTARÃO EM UM LABORATÓRIO LOCAL (LABORATÓRIO EMÍLIO AVELAR – CLÍNICA CENTERMED - ENDEREÇO: RUA DO FOGO, 400) ENTRE 07:00 E 08:00 DA MANHÃ SEGUINTE ÀS SESSÕES. Um cateter será inserido na veia antecubital, e uma amostra de sangue será coletada (min 0). Em seguida ingerem de 75 gramas de glicose em 300 ml de água e amostras de sangue são colhidas a cada 30 minutos até 120 minutos, totalizando 5 retiradas. As concentrações plasmáticas de glicose e insulina serão analisadas.

Critério de Inclusão:
A resistência à insulina tem sido relacionada com o aumento da lipotoxidade, consequência do aumento na quantidade de gordura corporal (Yazc & Sezer, 2017). Desta forma, os critérios de inclusão são indivíduos de ambos os sexos com obesidade (Índice de massa corporal – IMC > 30 kg/m2), e com obesidade central (circunferência da cintura > 102 cm em homens e > 88 cm em mulheres), com idade acima de 40 anos, COM MASSA CORPORAL ESTÁVEL (<3 KG) NOS ÚLTIMOS 3 MESES. OS PARTICIPANTES DEVEM AINDA SER CAPAZES DE REALIZAR ATIVIDADE FÍSICA, O QUE SERÁ AVALIADO PELAS RESPOSTAS DADAS DURANTE A ANAMNESE (ANEXO 1).

Critério de Exclusão:
OS CRITÉRIOS DE EXCLUSÃO SÃO INDIVÍDUOS COM SINAIS, SINTOMAS OU PRESENÇA DE DIABETES OU QUALQUER OUTRA DOENÇA METABÓLICA, DOENÇAS CARDIOVASCULARES, CEREBROVASCULARES, DOENÇAS RENAIS, DOENÇAS RESPIRATÓRIAS, E DOENÇAS OSTEOARTICULARES (RIEBE ET AL., 2015). ALÉM DISSO, SERÃO EXCLUÍDOS AQUELES QUE RELATEM USO DE QUALQUER MEDICAMENTO QUE POSSA INFLUENCIAR NOS RESULTADOS ESPERADOS E USO DE ESTEROIDES ANABOLIZANTES.

**Objetivo da Pesquisa:**

As informações aqui elencadas foram retiradas do arquivo Informações Básicas da Pesquisa (PB_Informações_Básicas_do_projeto_2014038, de 25/01/2023):

Objetivo Primário:
Objetivo Geral
O objetivo geral será investigar se o volume do exercício de força afeta a sensibilidade à insulina observada agudamente, isto é, a partir de uma única sessão, em adultos obesos.

Objetivo Secundário:
Objetivos específicos
OS OBJETIVOS ESPECÍFICOS SERÃO AVALIAR A INFLUÊNCIA AGUDA DE 7 EXERCÍCIOS DE GRANDES GRUPAMENTOS MUSCULARES REALIZADOS EM 3 OU 1 SÉRIES POR EXERCÍCIO, REALIZADAS ATÉ A FADIGA MOMENTÂNEA, EM ADULTOS OBESOS SOBRE:
1. A GLICOSE E A INSULINA DE JEJUM;
2. A RESISTÊNCIA À INSULINA PELO MODELO DE HOMEOSTASE DA RESISTÊNCIA À INSULINA (HOMA-IR);
3. A SENSIBILIDADE À INSULINA USANDO O ÍNDICE QUANTITATIVO DE VERIFICAÇÃO DE SENSIBILIDADE À INSULINA;5. O ÍNDICE DE SENSIBILIDADE À INSULINA DE MATSUDA;
6. O ÍNDICE DE SENSIBILIDADE À INSULINA DE CEDERHOLM ́S;
7. O ÍNDICE DE SENSIBILIDADE MUSCULAR À INSULINA;
8. O ÍNDICE DE SENSIBILIDADE À INSULINA ESTIMULADA POR GLICOSE; 9. O ÍNDICE DE DISPOSIÇÃO ORAL;
10. O ÍNDICE DE GUTT;
11. O ÍNDICE DE AVIGNON ET AL.;
12. ÍNDICE DE BELFIORE ET AL.;
13. ÍNDICE DE STUMVOLL ET AL.;
14. ÍNDICE DE MCAULEY ET AL.;
15. A ÁREA SOB A CURVA DE GLICOSE E DE INSULINA.

**Avaliação dos Riscos e Benefícios:**

As informações aqui elencadas foram retiradas do arquivo Informações Básicas da Pesquisa (PB_Informações_Básicas_do_projeto_2014038, de 25/01/2023):

Riscos:
Constrangimento. Existem riscos de desconforto e de constrangimento durante a aplicação da anamnese e preenchimento do recordatório alimentar. Serão minimizados pois o participante poderá deixar de responder a qualquer pergunta. Além disso, eles serão aplicados em sala reservada, de forma individualizada, onde apenas um membro da equipe e o participante estarão presentes. Identificação. Existe risco de identificação dos participantes na anamnese e recordatório alimentar. Serão minimizados pois apenas o pesquisador coordenador do projeto terá acesso às respostas aos documentos que ficarão guardados em sua sala de trabalho, trancados dentro de armário que apenas ele detém a chave, e após a finalização da pesquisa serão destruídos. Teste de tolerância oral à glicose. Existem riscos relacionados ao teste de tolerância oral à glicose. Em função da inserção do cateter na veia antecubital e da coleta de sangue, os principais riscos são fraqueza, ligeiro mal estar, sudorese (aumento na produção de suor), tontura, queda da pressão, hematoma (arroxeado), dor, infecção, e possibilidade de desconforto durante a coleta do sangue. Esses riscos serão minimizados pelos seguintes procedimentos: assepsia antes da coleta, colocação de compressa de gelo, utilização de materiais descartáveis, realização do procedimento por técnico treinado. A ingestão da solução de glicose pode trazer riscos relacionados a náuseas, tonteira, fraqueza, diarreia, taquicardia, tremores e sudorese (aumento na produção de suor). Esses riscos serão minimizados pela realização do teste em laboratório de análises especializado em procedimentos dessa natureza, onde há condições adequadas de lidar com qualquer intercorrência relatada acima, como deitar o participante em maca e a presença de toalete próximo. Medidas antropométricas. Existem riscos na realização das medidas antropométricas como constrangimento durante a medida da massa corporal, estatura e da circunferência da cintura. Para minimizar o risco de constrangimento, essa avaliação será feita apenas na presença de um membro da equipe em sala reservada. Eventos cardiovasculares. Para minimizar o risco de eventos cardio ou cerebrovasculares, os participantes responderão à anamnese e caso seja detectado sinais/sintomas de doenças cujas repercussões podem causar risco, eles serão excluídos do estudo. Ainda, caso eventos adversos ocorram durante as sessões os membros da equipe são treinados em como proceder com o atendimento de primeiros socorros. Exercício de força. Existem riscos durante a realização dos exercício de força relacionados a lesões músculo-esqueléticas. Para prevenir lesões, os participantes serão instruídos em relação à forma correta de se realizar os exercícios, realizarão sessões de familiarização e serão acompanhados em todas as sessões por profissional formado em Educação Física.

Benefícios:
Benefício direto: O teste de tolerância oral à glicose é um importante índice de diagnóstico de pré-diabetes e diabetes, portanto poderá servir para o participante conhecer melhor seu controle metabólico. A PARTICIPAÇÃO NESSA PESQUISA PODE MOTIVAR OS PARTICIPANTES E SE TORNAREM FISICAMENTE MAIS ATIVOS. Benefício indireto: OS RESULTADOS PUBLICIZADOS PROVENIENTES DA PESQUISA PODEM ALTERAR RECOMENDAÇÕES DE EXERCÍCIOS DE FORÇA PARA INDIVÍDUOS OBESOS QUE BUSCAM MELHORAR SUA SAÚDE.

**Comentários e Considerações sobre a Pesquisa:**

As informações aqui elencadas foram retiradas do arquivo Informações Básicas da Pesquisa (PB_Informações_Básicas_do_projeto_2014038, de 25/01/2023):

Metodologia de Análise de Dados:
COM OS RESULTADOS DO TESTE DE TOLERÂNCIA DE À GLICOSE, A INSULINA E GLICOSE NO MINUTO 0 (VALORES DE JEJUM) SERÃO UTILIZADAS PARA O CÁLCULO DA RESISTÊNCIA À INSULINA SERÁ CALCULADA A PARTIR DO MODELO DE HOMEOSTASE DA RESISTÊNCIA À INSULINA (HOMA-IR), USANDO A FÓRMULA GLICOSE (MMOL) X INSULINA ( μU/ML) ÷ 22,5 (MATTHEWS ET AL., 1985), E A SENSIBILIDADE À INSULINA USANDO O ÍNDICE QUANTITATIVO DE VERIFICAÇÃO DE SENSIBILIDADE À INSULINA (KATZ ET AL., 2000A). VÁRIOS ÍNDICES DE DERIVADOS DESTE TESTE (PATARRÃO ET AL., 2014A), COMO O ÍNDICE DE SENSIBILIDADE À INSULINA À GLICOSE ORAL (MARI ET AL., 2001A) ÍNDICE DE SENSIBILIDADE À INSULINA DE MATSUDA (MATSUDA & DEFRONZO, 1999A) E DE CEDERHOLM ́S (CEDERHOLM & WIBELL, 1990A), ÍNDICE DE SENSIBILIDADE MUSCULAR À INSULINA (ABDULGHANI ET AL., 2007A; MATOS ET AL., 2018), ÍNDICE DE SENSIBILIDADE À INSULINA ESTIMULADA POR GLICOSE (MALIN ET AL., 2013B), ÍNDICE DE DISPOSIÇÃO ORAL (ABDUL-GHANI ET AL., 2006; MIYAZAKI ET AL., 2008), ÍNDICE DE GUTT (GUTT ET AL., 2000), ÍNDICE DE AVIGNON ET AL. (COBELLI ET AL., 1987), ÍNDICE DE BELFIORE ET AL. (MONZILLO & HAMDY, 2003a), ÍNDICE DE STUMVOLL ET AL. (STUMVOLL ET AL., 2001a), ÍNDICE DE MCAULEY ET AL. (MCAULEY ET AL., 2001a). ALÉM DISSO, SERÁ CALCULADA A ÁREA SOB A CURVA DE GLICOSE E DE INSULINA USANDO O MÉTODO TRAPEZOIDAL (ISMAIL ET AL., 2019). Os dados serão expressos em média e desvio padrão, com intervalo de confiança em 95%. Para a análise da normalidade dos dados, realizaremos o teste de Shapiro-Wilk. E para os dados normalmente distribuídos será utilizada a análise de variância com uma fonte de variação (situação experimental). Caso observado efeito principal significativo, o post-hoc Tukey será usado. Para dados não paramétricos será utilizado o teste de Kruskal-Wallis, ou teste de Friedman ́s, quando necessário. O tamanho do efeito será calculado e interpretado da seguinte forma: 0,2 = baixo efeito, 0,5 = médio efeito, e maior que 0,8 = alto efeito (Ferguson, 2009; Sullivan & Feinn, 2012). O nível de significância será de 5%. O programa Prisma (GraphPad Software, San Diego, CA-USA – versão 9.3.1) será utilizado para análise dos resultados. Todo o trabalho de análise dos dados permanecerá cego até sua conclusão.

**Considerações sobre os Termos de apresentação obrigatória:**

Vide campo: “Conclusões e Pendências e Lista de Inadequações”

**Recomendações:**

Vide campo: “Conclusões e Pendências e Lista de Inadequações”

**Conclusões ou Pendências e Lista de Inadequações:**

Carta de anuência anexada. Projeto Aprovado.

**Considerações Finais a critério do CEP:**

- Segundo a Carta Circular no. 003/2011/CONEP/CNS, de 21/03/11, no momento da obtenção do TCLE, há obrigatoriedade de rubrica em todas as páginas do mesmo, pelo sujeito de pesquisa ou seu responsável e pelo pesquisador. O pesquisador responsável deverá apor sua assinatura na última página do referido termo.

- O Relatório final deverá ser apresentado ao CEP ao término do estudo. Considera-se como antiética a pesquisa descontinuada sem justificativa aceita pelo CEP que a aprovou.

- Caso haja quaisquer intercorrências durante a execução do projeto de pesquisa é de responsabilidade do pesquisador responsável comunicá-la através de uma emenda ao CEP via Plataforma Brasil. Considera-se como antiética a pesquisa com modificações em seu protocolo inicial previamente aprovado sem justificativa aceita pelo CEP que a aprovou.

O projeto atende aos preceitos éticos para pesquisas envolvendo seres humanos preconizados na Resolução 466/12 CNS.

**SITUAÇÃO DO PARECER:** APROVADO

**Necessita Apreciação da CONEP:** Não

DIAMANTINA, 02 de Fevereiro de 2023

**Assinado por:**

**FABIO LUIZ MENDONÇA MARTINS (Coordenador(a))**
