# Supplementary material for: The ASSIST trial: Acute effects of manipulating strength exercise volume on insulin sensitivity in obese adults: A protocol for a randomized controlled, crossover, clinical trial
Source: PLoS One. 2024 May 28;19(5):e0302480. doi: 10.1371/journal.pone.0302480 (PMC11132464; doi:10.1371/journal.pone.0302480)
Supplement: Attachment — Submitted filename: reply to Reviewers comments 23_12_18.docx [file pone.0302480.s006.docx]

We thank the reviewers for their time and effort on reviewing the protocol and provide below a point-by-point response to each of the comments **in bold letter**.

Reviewer #1: The reviewed paper is a description of the protocol of a planned study, aiming to assess strength exercise volume on insulin sensitivity.

It seems to be an interesting approach that may add additional information to the existing evidence regarding positive influence of exercise on the metabolism.

**We thank the reviewer for the comment.**

I would suggest only one measure that the authors could ask to the protocol, that is control but also use of heart rate during and after exercise as an additional parameter defining exercise. It may be that the same exercise may have different metabolic effect depending on how trained is a subject or patient.

**We appreciate this suggestion. However, resistance exercise intensity is not traditionally controlled by heart rate, nor is this response commonly assessed in resistance exercise studies. Also, in the present protocol we are interested in the metabolic, insulin-sensitizing effects of resistance exercise, not in the cardiovascular responses. Thus, we believe that adding this measurement will not add much to the protocol, or contribute to our aims, and may in fact burden the research personnel and participants. Therefore, we decided not to include heart rate measurement in this protocol. Nevertheless, if the reviewer strongly feels there is a rationale for this assessment, and thinks it would improve the quality of the protocol, we can consider including it.**

The other issue are the inclusion criteria. I would suggest to assess at least some laboratory parameters, like fasting plasma glucose(twice), OGGT , HbA1c and lipids – to answer whether participants have prediabetes, and to exclude diabetes (which is quite often undiagnosed in obese subjects) or high lipids. Maybe the authors (names and specialties have been blinded to me) would wish to consult a diabetologist/endocrinologist?

**We thank the reviewer for raising this important point and allowing us to address it. Type 2 diabetes mellitus (DM2) patients will be excluded from the study. Although we are not running pre-enrolment glucose assessments, in case DM2 is evident from the first OGTT they perform, participant will be excluded, following the reviewer’s suggestion and our exclusion criteria.**

Additionally, please justify the decision to perform the session between 8 and 9 pm the day before OGTT – why not earlier or later?

**Night-time exercise is common practice is “real-life” settings (e.g., fitness/health centers) as time constraints in modern life might hinder exercising earlier in the day. Also, studies on the effect of resistance exercise on insulin sensitivity have been conducted at this time** [1]**. Furthermore, data from a recent systematic review and meta-analysis showed no effect of night-time exercise on sleep quality or duration [2], suggesting night-time exercise can and should be performed. We added the following sentence in the manuscript under “Study type and design” to address the scheduling of our exercise sessions:**

**“*This time schedule for the sessions is ecologically valid, as exercise in the evening is commonly observed in fitness centers due to time constraints for exercising earlier during the day [54,55], and it has been shown that night-time exercise does not affect sleep duration or quality [54].”***

Minor issues: explaining that insulin sensitivity is an opposite to insulin resistance does not seem necessary to me.

**We have removed this from the abstract and introduction. We thank the reviewer for this suggestion.**

Please explain every abbreviation after its first appearance – like BRL, RM or others.

**Abbreviations were defined accordingly.**

Please define the time interval between the last familiarization session and study start.

**Under “Study type and design” we explain the timeline of the study:**

**“*After at least 4 days, they will carry out 3 experimental sessions, performed at random, separated by at least 4 (but no more than 28) washout days, […].”***

**Also, in figure 3 we highlight the interval between end of familiarization and beginning of the first session.**

Reviewer #2: Due to the inconclusive results of previous studies, the problem of the impact of particular strength training on insulin resistance remains unresolved.

**We thank the reviewer for this comment, but it is extremally important we clearly establish that the current study relates to the effects of an acute bout of resistance exercise, not training (several consecutive bouts over weeks/months/year). This distinction will set the scenario for addressing what appears to be one of the main points raised by the reviewer. To further clarify this point, we added to the “Introduction” the following sentence:**

**“*Interestingly, it has been reported that the training-induced improved on insulin sensitivity may be lost after as few as 4-6 days following the last training session [7,8], suggesting the positive effects of exercise on glycemic control can be largely attributed to the acute improvements observed in the hours-days after each exercise bout [9]. Notably, several studies point to the beneficial effects of a single acute bout of strength exercise on improving insulin sensitivity [10], and it has been reported that strength exercise sessions increase insulin sensitivity for up to 48 hours [11].”***

However, the research protocol raises my concerns:

– Too broad inclusion criteria for the study. It is observed in the clinical practice that an obese person may do not have insulin resistance. Moreover, the BMI criterion is insufficient because healthy, physically active people with developed muscle tissue may have a high BMI, but this does not mean that they are obese from a biochemical point of view. In my opinion, the study group should comprise people with confirmed laboratory indicators of insulin resistance, e.g. based on HOMA-IR.

**We thank the reviewer for raising this important point and we would happily address it. Including overweight/obese subjects regardless of their insulin resistance status for assessing the acute insulin-sensitizing effects of exercise is common practice in the literature. And this inclusion criteria sometimes leads to the inclusion in the same study of overweight/obese participants that do and do not present clinically established insulin resistance** [3–5]**, or even do and do not present impaired fasting glycemia** [6]**, to cite a few of many studies. Furthermore, it is an incomplete understanding of our inclusion criteria considering we would include participants only by assessing body mass index (although again, this is common practice in the relevant literature), as we clearly state that central obesity, assessed by waist circumference, is also to be used as a screening tool. Nevertheless, to make this point even more clear, and to add 2 more screening tools to characterize obesity and central adiposity, we added the following sentence under “Description of participants”:**

**“*In order to confirm the obesity and central adiposity statuses of the participants, dual-energy X-ray absorptiometry will be used to assess fat percentage and visceral fat mass (see details below in Anthropometric measurements), and participants will be included only if both indexes are >90th percentile for age and sex [37].”***

**Also, many studies were cited throughout the paper to show that within the type 2 diabetes mellitus spectrum, and regardless of the disease status (from healthy to obesity to insulin resistance to prediabetes to type 2 diabetes mellitus) the effects of resistance exercise on improving glucose metabolism have been observed.**

**Finally, we acknowledge this potential source of variation in the “Weakness” section within the “Discussion” chapter:**

**“*Furthermore, individuals with potentially different levels of insulin resistance might be included in this study, and greater exercise-induced improvement in insulin resistance has been observed in individuals with higher levels of baseline insulin resistance [28,102]. Thus, this factor can also increase results variability. However, it should be pointed out that studying overweight/obese subjects regardless of their insulin resistance or fasting glucose impairment status is commonplace in the related literature [8,103–105]. Nevertheless, the fact that participants will be their own control minimizes this potential source of variability. Moreover, individual results will be presented and discussed accordingly should individual levels of insulin resistance affect average group results. Also, DM2 patients will be excluded, following our exclusion criteria.”***

**Therefore, we would like to keep our inclusion criteria as it stands, and hope the above clarification helps on settling this issue.**

– Measuring insulin resistance based on the OGTT result seems to represent imprecise methodology. Basing basically the entire study on this parameter seems to be an oversimplification and lead to false conclusions.

**We thank the reviewer for addressing this point. We expanded on the explanation as to why will be used, based on the relevant literature under “Strengths” within the “Discussion” chapter:**

**“*Finally, although the OGTT might not be the gold-standard tool to assess insulin sensitivity compared to the euglycemic-hyperinsulinemic clamp (EHC), ingesting 75 grams of glucose is considered to be a more physiological stimulus compared the supraphysiological insulin levels during EHC [90]. Moreover, the insulin sensitivity indexes that will be calculated from OGTT in the present study show high correlation (r=0.61 and 0.96) [91] with EHC results. Also, OGTT is considered reliable and consistent for estimating insulin sensitivity over consecutive days [92]. Last, but not the least, the majority of studies that assessed the acute effects of a strength exercise session on insulin sensitivity employed OGTT as the assessment tool [28,50,53,93–101].*”**

**Thus, we respectfully disagree with impression the reviewer has on the OGTT being imprecise and that its results can lead to false conclusions, as the literature presented in the sentence above shows the exact opposite, and majority of studies on the effects of resistance exercise on glucose metabolism were performed using this technique.**

– Performing a glucose tolerance test several hours after exercise seems inappropriate, because "acute" processes related to glucose utilization by the tissues are still taking place at that time. Thus, the determined parameters will reflect the body's acute response to increased glucose utilization rather than the possible increase in insulin sensitivity. It seems reasonable to increase the time interval between the training session and blood drawing and measure the true change in insulin sensitivity,

**We thank the reviewer for addressing this point, and would like to make it clear that the present protocol was designed to investigate the *acute* (one bout), not *chronic* (training, several bouts) effects of resistance exercise. This is based on the evidence that the exercise training effects on improved insulin sensitivity are not a true training effect, but the lingering acute effect of the last exercise session** [5,7]**, which suggests that understanding the acute effects of exercise on insulin sensitivity is of great clinical importance. Therefore, the proximity between the exercise bout and the OGTT is by design, not a methodological limitation.**

– Small number of participants in the study. Such sample size does not ensure that analyzed parameters will reach adequate power. In the power analysis of the test only the Matsuda insulin sensitivity index was used.

**To address this potential anticipated limitation, we had previously calculated sample size based on all insulin sensitivity indexes we will assess (i.e., the oral glucose insulin sensitivity index [8], the Matsuda insulin sensitivity index [9] and Cederholm's index [10], muscle insulin sensitivity index [11,12], glucose-stimulated insulin sensitivity index [13], oral disposition index [14,15], Gutt index [16], Avignon et al. [17], Belfiore et al. index [18], Stumvoll et al. index [19], and McAuley et al. index [20]), and when we used the Matsuda index the number of participants needed was the greatest. That is why we chose this index tom calculate sample size. Had we based the calculation exclusively on another index, sample size would have been smaller, and the chance of the study being underpowered for the other indexes would increase. Finally, an inspection of the sample size from the literature pertaining to the effects of resistance exercise on insulin sensitivity [1,21–36] reveals an average of 15.95 participants, with a huge variation (SD = 8.23). Therefore, we are confident that we were diligent when calculating our sample size, as we used the index that gave us the greatest number of subjects needed, and the calculated sample size is within the sample sizes used in studies that addressed similar research problems.**

– Presented research program is not innovative.

**Once again, we respectfully disagree with the impression of the reviewer. In a thorough literature review, we were unable to find one single study that had similar study design, especially when considering the scientific rigor we are employing in our protocol (adhering the Spirit guidelines, blinding of data analyst, blinding of statistician, etc.). If the reviewer is aware of such a study from which the current protocol does not differ, or innovates from, we kindly ask for the reference, and we would gladly read this paper, and discuss or even change our protocol accordingly.**

The subject matter of the work is more consistent with journals on physiotherapy or exercise physiology.

**In the PlosOne website, under the scope of the journal, one reads:**

**“PLOS ONE welcomes original research submissions from the natural sciences, medical research, engineering, as well as the related social sciences and humanities, including:**

**Protocols, including Lab Protocols that describe verified methodologies and Study Protocols that describe detailed plans for research projects.**

**[authors underlined the text above].**

**Therefore, we the authors were somewhat surprised with the above statement by the reviewer, since at no moment PlosOne guidelines prohibit or exclude papers from one particular area. Quite the opposite: it welcomes papers from medical research and study protocols. In fact, a quick search in the PlosOne website reveals many study protocols that relate to similar medical areas [37,38] the current protocol falls into. Thus, we do not believe a paper in PlosOne should be rejected or even criticized based on the area it more closely relates to, especially if the paper falls within the scope of the journal, and previous similar papers were published recently in the journal.**

References

1.	Tong TK, Kong Z, Shi X, Shi Q. Comparable Effects of Brief Resistance Exercise and Isotime Sprint Interval Exercise on Glucose Homeostasis in Men. J Diabetes Res. 2017;2017. doi:10.1155/2017/8083738

2.	Kahn M, Korhonen T, Leinonen L, Martinmaki K, Kuula L, Pesonen A-K, et al. Is It Time We Stop Discouraging Evening Physical Activity? New Real-World Evidence From 150,000 Nights. Front Public Health. 2021;9. doi:10.3389/fpubh.2021.772376

3.	Whyte LJ, Ferguson C, Wilson J, Scott RA, Gill JMR. Effects of single bout of very high-intensity exercise on metabolic health biomarkers in overweight/obese sedentary men. Metabolism. 2013;62: 212–219. doi:10.1016/j.metabol.2012.07.019

4.	Durrer C, Robinson E, Wan Z, Martinez N, Hummel ML, Jenkins NT, et al. Differential Impact of Acute High-Intensity Exercise on Circulating Endothelial Microparticles and Insulin Resistance between Overweight/Obese Males and Females. PLoS One. 2015;10: e0115860. doi:10.1371/journal.pone.0115860

5.	Ryan BJ, Schleh MW, Ahn C, Ludzki AC, Gillen JB, Varshney P, et al. Moderate-Intensity Exercise and High-Intensity Interval Training Affect Insulin Sensitivity Similarly in Obese Adults. J Clin Endocrinol Metab. 2020;105: e2941–e2959. doi:10.1210/clinem/dgaa345

6.	Little JP, Jung ME, Wright AE, Wright W, Manders RJF. Effects of high-intensity interval exercise versus continuous moderate-intensity exercise on postprandial glycemic control assessed by continuous glucose monitoring in obese adults. Applied Physiology, Nutrition, and Metabolism. 2014;39: 835–841. doi:10.1139/apnm-2013-0512

7.	Dela F, Larsen JJ, Mikines KJ, Ploug T, Petersen LN, Galbo H. Insulin-Stimulated Muscle Glucose Clearance in Patients With NIDDM: Effects of One-Legged Physical Training. Diabetes. 1995;44: 1010–1020. doi:10.2337/diab.44.9.1010

8.	Mari A, Pacini G, Murphy E, Ludvik B, Nolan JJ. A Model-Based Method for Assessing Insulin Sensitivity From the Oral Glucose Tolerance Test. Diabetes Care. 2001;24: 539–548. doi:10.2337/diacare.24.3.539

9.	Matsuda M, DeFronzo RA. Insulin sensitivity indices obtained from oral glucose tolerance testing: comparison with the euglycemic insulin clamp. Diabetes Care. 1999;22: 1462–1470. doi:10.2337/diacare.22.9.1462

10.	Cederholm J, Wibell L. Insulin release and peripheral sensitivity at the oral glucose tolerance test. Diabetes Res Clin Pract. 1990;10: 167–175. doi:10.1016/0168-8227(90)90040-Z

11.	Abdul-Ghani MA, Matsuda M, Balas B, DeFronzo RA. Muscle and Liver Insulin Resistance Indexes Derived From the Oral Glucose Tolerance Test. Diabetes Care. 2007;30: 89–94. doi:10.2337/dc06-1519

12.	Matos MA de, Vieira DV, Pinhal KC, Lopes JF, Dias-Peixoto MF, Pauli JR, et al. High-Intensity Interval Training Improves Markers of Oxidative Metabolism in Skeletal Muscle of Individuals With Obesity and Insulin Resistance. Front Physiol. 2018;9. doi:10.3389/fphys.2018.01451

13.	Malin SK, Hinnerichs KR, Echtenkamp BG, Evetovich TK, Engebretsen BJ. Effect of adiposity on insulin action after acute and chronic resistance exercise in non-diabetic women. Eur J Appl Physiol. 2013;113: 2933–2941. doi:10.1007/s00421-013-2725-5

14.	Abdul-Ghani MA, Tripathy D, DeFronzo RA. Contributions of β-Cell Dysfunction and Insulin Resistance to the Pathogenesis of Impaired Glucose Tolerance and Impaired Fasting Glucose. Diabetes Care. 2006;29: 1130–1139. doi:10.2337/dc05-2179

15.	Miyazaki Y, Akasaka H, Ohnishi T, Saitoh , Shigeyuki, DeFronzo RA, Shimamoto K. Differences in Insulin Action and Secretion, Plasma Lipids and Blood Pressure Levels between Impaired Fasting Glucose and Impaired Glucose Tolerance in Japanese Subjects. Hypertension Research. 2008;31: 1357–1363. doi:10.1291/hypres.31.1357

16.	Gutt M, Davis CL, Spitzer SB, Llabre MM, Kumar M, Czarnecki EM, et al. Validation of the insulin sensitivity index (ISI0,120): comparison with other measures. Diabetes Res Clin Pract. 2000;47: 177–184. doi:10.1016/S0168-8227(99)00116-3

17.	Cobelli C, Mari A, Ferrannini E. Non-steady state: error analysis of Steele’s model and developments for glucose kinetics. American Journal of Physiology-Endocrinology and Metabolism. 1987;252: E679–E689. doi:10.1152/ajpendo.1987.252.5.E679

18.	Monzillo LU, Hamdy O. Evaluation of Insulin Sensitivity in Clinical Practice and in Research Settings. Nutr Rev. 2003;61: 397–412. doi:10.1301/nr.2003.dec.397-412

19.	Stumvoll M, van Haeften T, Fritsche A, Gerich J. Oral Glucose Tolerance Test Indexes for Insulin Sensitivity and Secretion Based on Various Availabilities of Sampling Times. Diabetes Care. 2001;24: 796–797. doi:10.2337/diacare.24.4.796

20.	McAuley KA, Williams SM, Mann JI, Walker RJ, Lewis-Barned NJ, Temple LA, et al. Diagnosing Insulin Resistance in the General Population. Diabetes Care. 2001;24: 460–464. doi:10.2337/diacare.24.3.460

21.	Koopman R, Manders RJF, Zorenc AHG, Hul GBJ, Kuipers H, Keizer HA, et al. A single session of resistance exercise enhances insulin sensitivity for at least 24 h in healthy men. Eur J Appl Physiol. 2005;94: 180–187. doi:10.1007/s00421-004-1307-y

22.	Fluckey JD, Hickey MS, Brambrink JK, Hart KK, Alexander K, Craig BW. Effects of resistance exercise on glucose tolerance in normal and glucose-intolerant subjects. J Appl Physiol. 1994;77: 1087–1092. doi:10.1152/jappl.1994.77.3.1087

23.	CHAPMAN J, GARVIN AW, WARD A, CARTEE GD. Unaltered insulin sensitivity after resistance exercise bout by postmenopausal women. Med Sci Sports Exerc. 2002;34: 936–941. doi:10.1097/00005768-200206000-00005

24.	Gordon BA, Fraser SF, Bird SR, Benson AC. Insulin sensitivity not modulated 24 to 78 h after acute resistance exercise in type 2 diabetes patients. Diabetes Obes Metab. 2013;15: 478–480. doi:10.1111/dom.12057

25.	BITTEL AJ, BITTEL DC, MITTENDORFER B, PATTERSON BW, OKUNADE AL, ABUMRAD NA, et al. A Single Bout of Premeal Resistance Exercise Improves Postprandial Glucose Metabolism in Obese Men with Prediabetes. Med Sci Sports Exerc. 2021;53: 694–703. doi:10.1249/MSS.0000000000002538

26.	van Dijk J-W, Manders RJF, Tummers K, Bonomi AG, Stehouwer CDA, Hartgens F, et al. Both resistance- and endurance-type exercise reduce the prevalence of hyperglycaemia in individuals with impaired glucose tolerance and in insulin-treated and non-insulin-treated type 2 diabetic patients. Diabetologia. 2012;55: 1273–1282. doi:10.1007/s00125-011-2380-5

27.	Black LE, Swan PD, Alvar BA. Effects of Intensity and Volume on Insulin Sensitivity During Acute Bouts of Resistance Training. J Strength Cond Res. 2010;24: 1109–1116. doi:10.1519/JSC.0b013e3181cbab6d

28.	Brown EC, Franklin BA, Regensteiner JG, Stewart KJ. Effects of single bout resistance exercise on glucose levels, insulin action, and cardiovascular risk in type 2 diabetes: A narrative review. J Diabetes Complications. 2020;34: 107610. doi:10.1016/j.jdiacomp.2020.107610

29.	Reed ME, Ben-Ezra V, Biggerstaff KD, Nichols DL. The Effects of Two Bouts of High- and Low-Volume Resistance Exercise on Glucose Tolerance in Normoglycemic Women. J Strength Cond Res. 2012;26: 251–260. doi:10.1519/JSC.0b013e318218dea3

30.	MORENO-CABAÑAS A, ORTEGA JF, MORALES-PALOMO F, RAMIREZ-JIMENEZ M, ALVAREZ-JIMENEZ L, MORA-RODRIGUEZ R, et al. One Bout of Resistance Training Does Not Enhance Metformin Actions in Prediabetic and Diabetic Individuals. Med Sci Sports Exerc. 2022;54: 1043–1050p. doi:https://doi.org/10.1249/MSS.0000000000002889

31.	Monroe JC, Naugle KM, Naugle KE. Effect of Acute Bouts of Volume-Matched High-Intensity Resistance Training Protocols on Blood Glucose Levels. J Strength Cond Res. 2020;34: 445–450. doi:10.1519/JSC.0000000000002994

32.	Malin SK, Hinnerichs KR, Echtenkamp BG, Evetovich TK, Engebretsen BJ. Effect of adiposity on insulin action after acute and chronic resistance exercise in non-diabetic women. Eur J Appl Physiol. 2013;113: 2933–2941. doi:10.1007/s00421-013-2725-5

33.	Andersen E, Høstmark AT. Effect of a Single Bout of Resistance Exercise on Postprandial Glucose and Insulin Response the Next Day in Healthy, Strength-Trained Men. The Journal of Strength and Conditioning Research. 2007;21: 487. doi:10.1519/R-20105.1

34.	Fenicchia LM, Kanaley JA, Azevedo JL, Miller CS, Weinstock RS, Carhart RL, et al. Influence of resistance exercise training on glucose control in women with type 2 diabetes. Metabolism. 2004;53: 284–289. doi:10.1016/j.metabol.2003.10.007

35.	Gordon BA, Fraser SF, Bird SR, Benson AC. Insulin sensitivity in response to a single resistance exercise session in apparently healthy individuals. J Endocrinol Invest. 2012;35: 665–669. doi:10.3275/7972

36.	Luebbers PE, Potteiger JA, Warren BJ, Clore JN, Jennings C, Bond DS. GLUCOSE UPTAKE AFTER RESISTANCE TRAINING OF DIFFERENT INTENSITIES BUT OF EQUAL WORK VOLUME. Available: www.nsca-jscr.org

37.	Pearson AG, Macnaughton LS, Hind K. Sex differences in the impact of resistance exercise load on muscle damage: A protocol for a randomised parallel group trial. PLoS One. 2022;17: e0275221. doi:10.1371/journal.pone.0275221

38.	Silva H de J, Fonseca LS, Mascarenhas RO, Miranda JP de, Almeida PA, Souza MB, et al. The ESCAPE trial for older people with chronic low back pain: Protocol of a randomized controlled trial. PLoS One. 2022;17: e0266613. doi:10.1371/journal.pone.0266613
