# Supplementary material for: The ASSIST trial: Acute effects of manipulating strength exercise volume on insulin sensitivity in obese adults: A protocol for a randomized controlled, crossover, clinical trial
Source: PLoS One. 2024 May 28;19(5):e0302480. doi: 10.1371/journal.pone.0302480 (PMC11132464; doi:10.1371/journal.pone.0302480)
Supplement: Attachment — Submitted filename: Response to Reviewers_24_03_07.docx [file pone.0302480.s007.docx]

Editor comments.

**We thank the editor for giving us the opportunity to reply to the reviewers’ comments. Our replies are in bold following each comment.**

Journal Requirements:

1. Please ensure that your manuscript meets PLOS ONE's style requirements, including those for file naming. The PLOS ONE style templates can be found at
<https://journals.plos.org/plosone/s/file?id=wjVg/PLOSOne_formatting_sample_main_body.pdf> and
<https://journals.plos.org/plosone/s/file?id=ba62/PLOSOne_formatting_sample_title_authors_affiliations.pdf>

**We changed the format of sections to match the journal’s requirements.**

2. We note that the grant information you provided in the ‘Funding Information’ and ‘Financial Disclosure’ sections do not match.When you resubmit, please ensure that you provide the correct grant numbers for the awards you received for your study in the ‘Funding Information’ section.

**We doubled edited the funding information in the main text to match the one provided in the submission platform. Also, we removed the institution support (basically equipment and space) to the acknowledgments section, as the institutional where data were collected did not provide financial support.**

3. Thank you for stating the following financial disclosure:

"NO - The funders did not and will not have a role in study design, data collection and analysis, decision to publish, or preparation of the manuscript."
At this time, please address the following queries:
a) Please clarify the sources of funding (financial or material support) for your study. List the grants or organizations that supported your study, including funding received from your institution.
b) State what role the funders took in the study. If the funders had no role in your study, please state: “The funders had no role in study design, data collection and analysis, decision to publish, or preparation of the manuscript.”
c) If any authors received a salary from any of your funders, please state which authors and which funders.
d) If you did not receive any funding for this study, please state: “The authors received no specific funding for this work.”
Please include your amended statements within your cover letter; we will change the online submission form on your behalf.

**We changed the text accordingly in the “Funding and role of funding source” section to:**

**This study is supported by the Conselho Nacional de Desenvolvimento Científico e Tecnológico (CNPQ: Grant#407975/2018-7 and #402091/2021-3) and by the Fundação de Amparo à Pesquisa do Estado de Minas Gerais (FAPEMIG: Grant#APQ-00008-22). The funders had no role in study design, data collection and analysis, decision to publish, or preparation of the manuscript.**

4. When completing the data availability statement of the submission form, you indicated that you will make your data available on acceptance. We strongly recommend all authors decide on a data sharing plan before acceptance, as the process can be lengthy and hold up publication timelines. Please note that, though access restrictions are acceptable now, your entire data will need to be made freely accessible if your manuscript is accepted for publication. This policy applies to all data except where public deposition would breach compliance with the protocol approved by your research ethics board. If you are unable to adhere to our open data policy, please kindly revise your statement to explain your reasoning and we will seek the editor's input on an exemption. Please be assured that, once you have provided your new statement, the assessment of your exemption will not hold up the peer review process.

**We changed the text in the submission platform and in S2_Table to:**

**“Deidentified research data will be made publicly available when the study is completed and published.”**

Please review your reference list to ensure that it is complete and correct. If you have cited papers that have been retracted, please include the rationale for doing so in the manuscript text, or remove these references and replace them with relevant current references. Any changes to the reference list should be mentioned in the rebuttal letter that accompanies your revised manuscript. If you need to cite a retracted article, indicate the article’s retracted status in the References list and also include a citation and full reference for the retraction notice.

**We are not aware of any citation included that was retracted.**

Reviewers' comments:

Reviewer #2: I have no comments, the authors made necessary amendments and clarified doubtful issues. This study may be valuable contribution to our understanding of insulin sensitivity.

**We appreciate the compliment, and thank the reviewer for their time and effort in reviewing this study.**

Reviewer #3: Great job on this study design, below are a few comments:

**We appreciate the compliment, and thank the reviewer for their time and effort in reviewing this study.**

- personally I would suggest adjusting the title and making it more specific. Make sure it is clear that the study is focusing on an acute period, not long term. Stating a questions like this is too general and does not represent what you are investigating.

**We thank the reviewer for this comment. In fact, the term “acute” is the very first word in the title, so respectfully, we do not see the need to restate that the study is acute in the title once more. Should the reviewer have a suggestion for changing the title, we can certainly change it accordingly.**

- Currently there is a large difference in exercise volume. Consider adding one group that does 2 sets so you have one group that does 14 sets. This allows you to make a firmer statement.

**This large difference in exercise volume is intentional. Although we agree that a dose-response study on the effect of resistance exercise on insulin sensitivity is warranted, our rationale for the present study relates to time-commitment, as stated in the introduction. As sedentary, obese individuals might feel demotivated to perform a traditional, high-volume resistance exercise session due to time constraints and low self-efficacy, our idea was to drastically reduce exercise volume, and by default, session duration, in order to assess whether a shorter session, with lower volume would still be effective in promoting beneficial effects on insulin sensitivity. Thus, our aims in this stufy do not relate to assessing a dose-response relationship. On top of that, another situation with 14 sets would increase the burden on the subjects, potentially reducing participation adherence.**

**To address this important point raised by the reviewer, we included the following sentence in the “weakness” subsection within the Discussion section:**

**“Finally, per our study design, we will assess two significantly different strength exercise volumes, and by default time commitment, as lack of time is usually listed as one of the main reasons not to adhere to exercise [31]. Thus, our study design is not intended to explore a potential dose-response relationship between strength exercise volume and improvements in insulin sensitivity, although this approach would bear practical implications.”**

- It is not fully clear to me ppwhat this study will ad on top of the already available studies. Black et al Is mentioned in the manuscript and I am not convinced that the current study will add a lot of information on to this paper. It looks to me as a study designed to confirm what is already been done.

**We thank the reviewer for the opportunity to make the case for our study design and outline how it differs from previous literature, specially form Black et al [1]. There are many points that the current study design differs, and in our opinion, improves on the Black’s et al’s study. First, authors assessed women with childbearing capacity, but did not report, nor discussed menstrual cycle phase, different from our study design. Second, they only performed one familiarization session, which was followed by a 5-RM strength test session. Having previously strength exercise naïve subjects perform only one familiarization session before engaging in a quite demanding strength test session is not best practice, as true familiarization most likely was not attained. Not being fully familiarized with the resistance exercises is a big concern when assessing insulin sensitivity, as unaccustomed strength exercise can lead to worsening of insulin sensitivity [2–4]. Our current study design anticipates at least 4 familiarization sessions before the strength tests session to ensure participants are fully familiarized with the research protocol. Third, the study of Black et al used the glucose and insulin data from the morning before the exercise sessions as baseline, when best practice on clinical trials dictates a control (sham) day should be employed, as will be done in the present study. Fourth, that study assessed insulin sensitivity only by calculating HOMA-IR. Not only HOMA-IR is a much more limited tool to address insulin sensitivity compared to OGTT-derived indexes[5], but it is actually a measure of insulin resistance, more specifically, liver insulin resistance[6], and insulin sensitivity is not the mere opposite of insulin resistance [7]. In the present study, we will assess HOMA-IR, but most importantly, OGTT-derived indexes will provide us the opportunity to tease out changes in muscle insulin sensitivity and liver insulin resistance as they change in response to a strength exercise bout. Fifth, Black et al do not report any actions taken toward blinding glucose and insulin measurements, nor the statistical analysis, different from what we propose to do. Sixth, and perhaps most importantly, Black et al do not describe the degree of effort (or proximity to failure) in each set. This is very important because as stated in our introduction and discussion, the degree of effort is regarded as a factor that affects the effect on strength exercise on glucose and insulin responses [8]. Although degree of effort was not reported, per Black et al’s design, it can be assumed that in the “high-volume” situations the degree of effort was higher, as fatigue would have accumulated with multiple sets. Our design anticipates participants will perform all sets in both exercise sessions to failure, thus a true volume effect will be isolated from this important confounding variable.**

**Altogether, we believe the current design of our study improves on the design reported by Black et al and we believe the data generated by our study will stem from a much higher methodological quality.**

**To address Black et al’s limitations, we added the following sentence:**

**“Black et al [29] evaluated 8 exercises performed with 1 or 4 sets, either at 65% 1RM (12-15 reps) or 85% 1RM (6 to 8 reps). The authors observed improvements in insulin resistance in all protocols; but, protocols with single sets showed less effect. However, in that study, the degree of effort in each set was not reported and arguably higher in the high-volume conditions, and as this parameter is suggested as a determinant in the improvement of insulin sensitivity [25], one cannot exclude the possibility this factor played a role in the results observed. Furthermore, Black et al [29] assessed insulin resistance by the homeostasis model assessment of insulin resistance (HOMA-IR), which is more limited than other methods, such as OGTT-derived indexes [30], and more closely reflects hepatic insulin resistance [31].”**

- The cross over model is great!

**We appreciate the compliment.**

- It is important to keep the time in between days similar within and between the participants. A range of 4 to 28 days between trials is now given which to me seems like a lot of variance.

**As stated in the “Study type and design” section, if the interval between sessions are to be more than 21 days apart, participants will perform another familiarization session 1 to 2 weeks preceding the following session, thus the interval between a familiarization and a study session will be 4 to 14 days. We had to do this, because as women with childbearing potential can be enrolled, and we aim to assess them in the follicular phase of the menstrual cycle, we had to figure out a way to keep them familiarized to the study protocol, but at the same avoiding a training effect. It has been shown that even once a week strength training can lead to improvement in strength [9,10]. So, we could not have them familiarized once a week (nor more frequent than that), because this could lead to a training effect. To address this point, we included the following statement to “Study type and design”:**

**“The interval between experimental sessions will be recorded and reported in the final manuscript, and discussed accordingly.”**

- The 8RM for all 7 exercises in 1 day seems like a lot. How are you going to ensure that the participant is not fatigued at the last exercise? 2 minutes of rest is stated, is this sufficient?

**Performing strength tests in all seven exercises in the same strength session is intentional. As participants will perform the seven exercises in the exercise sessions back-to-back, we wanted them to be tested in the same fashion. Although this design will probably lead to some fatigue carryover, we believe it more closely aligns with the practical applications of our study, and mimics the actual fatigue participants will have to endure during the exercise sessions. Also, this is not a training study, and the measurement of strength in the present protocol will only serve to prescribe the load during the exercise sessions. Finally, in both exercise sessions, they will lift the exact same load, so even if we do not find a “real” 8-RM in every exercise, this will have no impact our study design or results’ interpretation.**

**To address this, we added the following statement to the “Strength tests” subsection:**

**“Because during the high- and low-volume experimental sessions participants will perform all seven exercises sequentially, and fatigue will likely accumulate as the sessions progresses, we decided to have their 8-RM tested in the same fashion, in order to mimic their anticipated effort. This means a true 8-RM might not be recorded, especially for exercises tested later in the strength test session. However, we believe this will not interfere with our study design and results interpretation, as exactly the same load will be prescribed for the exercise experimental sessions.”**

- It is suggested to base your exclusion criteria on baseline insulin resistance. Personally I would suggest picking some cut off values to make the study more specific and to prevent outliers in your data.

**Respectfully, we believe we have already addressed this point in the “Weaknesses” subsection within the discussion section, as it reads below:**

**“Furthermore, individuals with potentially different levels of insulin resistance might be included in this study, and greater exercise-induced improvement in insulin resistance has been observed in individuals with higher levels of baseline insulin resistance [29,102]. Thus, this factor can also increase results variability. However, it should be pointed out that studying overweight/obese subjects regardless of their insulin resistance or fasting glucose impairment status is commonplace in the related literature [8,103–105]. Nevertheless, the fact that participants will be their own control minimizes this potential source of variability. Moreover, individual results will be presented and discussed accordingly should individual levels of insulin resistance affect average group results. Also, DM2 patients will be excluded, following our exclusion criteria.”**

- A lot of details are giving in the explanation of the exercise and diet for all the conditions. This part seems well thought out to me. Good job.

**We appreciate the compliment.**

- I would suggest going over the structure of some sentences again to make sure there is a nice flow in the manuscript.

**We appreciate this comment, and have performed a thorough revision in the entire manuscript, with special attention to the introduction, as now in hindsight we believe was longer than necessary. The specific changes can be tracked in the track changes version.**

References

1.	Black LE, Swan PD, Alvar BA. Effects of intensity and volume on insulin sensitivity during acute bouts of resistance training. J Strength Cond Res. 2010;24: 1109–1116. doi:10.1519/JSC.0b013e3181cbab6d

2.	Chen TC, Huang M-J, Lima LCR, Chou T-Y, Wang H-H, Tu J-H, et al. Changes in Insulin Sensitivity and Lipid Profile Markers Following Initial and Secondary Bouts of Multiple Eccentric Exercises. Front Physiol. 2022;13. doi:10.3389/fphys.2022.917317

3.	Jimenez C, Santiago M, Sitler M, Boden G, Homko C. Insulin-sensitivity response to a single bout of resistive exercise in type 1 diabetes mellitus. J Sport Rehabil. 2009;18: 564–571. doi:http://dx.doi.org/10.1123/jsr.18.4.564

4.	Gonzalez JT, Barwood MJ, Goodall S, Thomas K, Howatson G. Alterations in whole-body insulin sensitivity resulting from repeated eccentric exercise of a single muscle group: A pilot investigation. Int J Sport Nutr Exerc Metab. 2015;25: 405–410. doi:10.1123/ijsnem.2014-0211

5.	Patarrão RS, Wayne Lautt W, Paula Macedo M. Assessment of methods and indexes of insulin sensitivity. Revista Portuguesa de Endocrinologia, Diabetes e Metabolismo. 2014;9: 65–73. doi:10.1016/j.rpedm.2013.10.004

6.	Qureshi K, Clements RH, Saeed F, Abrams GA. Comparative Evaluation of Whole Body and Hepatic Insulin Resistance Using Indices from Oral Glucose Tolerance Test in Morbidly Obese Subjects with Nonalcoholic Fatty Liver Disease. J Obes. 2010;2010: 1–7. doi:10.1155/2010/741521

7.	Ronald Kahn C. Insulin resistance, insulin insensitivity, and insulin unresponsiveness: A necessary distinction. Metabolism. 1978;27: 1893–1902. doi:10.1016/S0026-0495(78)80007-9

8.	Brown EC, Franklin BA, Regensteiner JG, Stewart KJ. Effects of single bout resistance exercise on glucose levels, insulin action, and cardiovascular risk in type 2 diabetes: A narrative review. J Diabetes Complications. 2020;34: 107610. doi:10.1016/j.jdiacomp.2020.107610

9.	DiFrancisco-Donoghue J, Werner W, Douris PC, McKenna RF. Comparison of once-weekly and twice-weekly strength training in older adults * Commentary. Br J Sports Med. 2006;41: 19–22. doi:10.1136/bjsm.2006.029330

10.	Taaffe DR, Duret C, Wheeler S, Marcus R. Once‐Weekly Resistance Exercise Improves Muscle Strength and Neuromuscular Performance in Older Adults. J Am Geriatr Soc. 1999;47: 1208–1214. doi:10.1111/j.1532-5415.1999.tb05201.x
